# Supplementary material for: Tuning relaxation and nonlinear upconversion of valley-exciton-polaritons in a monolayer semiconductor
Source: Nat Commun. 2025 Nov 3;16:9700. doi: 10.1038/s41467-025-65737-5 (PMC12583619; doi:10.1038/s41467-025-65737-5)
Supplement: Supplementary file 1 — Supplementary Information [file 41467_2025_65737_MOESM1_ESM.pdf]

Tuning relaxation and nonlinear upconversion of valley-exciton-polaritons in a monolayer semiconductor

Hangyong Shan[1], Jamie M. Fitzgerald[2], Roberto Rosati[2], Gilbert Leibeling[3,4,5], Kenji Watanabe[6], Takashi Taniguchi[7], Seth Ariel Tongay[8], Falk Eilenberger[3,4,5], Martin Esmann[1], Sven Höfling[9], Ermin Malic[2] and Christian Schneider[1,*]

[1]Institute of Physics, Carl von Ossietzky University Oldenburg, 26129 Oldenburg, Germany
[2]Department of Physics, Philipps-Universität Marburg, 35032 Marburg, Germany
[3]Institute of Applied Physics, Abbe Center of Photonics, Friedrich Schiller University Jena, 07745 Jena, Germany
[4]Fraunhofer-Institute for Applied Optics and Precision Engineering IOF, 07745 Jena, Germany
[5]Max-Planck-School of Photonics, 07745 Jena, Germany
[6]Research Center for Electronic and Optical Materials, National Institute for Materials Science, 1-1 Namiki, Tsukuba 305-0044, Japan
[7]Research Center for Materials Nanoarchitectonics, National Institute for Materials Science, 1-1 Namiki, Tsukuba 305-0044, Japan
[8]School for Engineering of Matter, Transport, and Energy, Arizona State University, Tempe, Arizona 85287, USA
[9]Julius-Maximilians-Universität Würzburg, Physikalisches Institut and Würzburg-Dresden Cluster of Excellence ct.qmat, Lehrstuhl für Technische Physik, Am Hubland, 97074 Würzburg, Germany

[*]Corresponding author: christian.schneider@uni-oldenburg.de

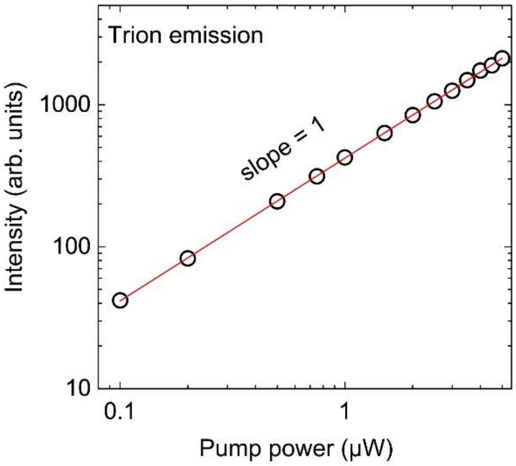

Fig. S1 Pump power dependent PL intensity of trions for the $MoSe_2$/GaInP/Bottom DBRs sample. The sample is excited with a continuous wave (CW) 532 nm laser. This linear carrier relaxation is in distinct contrast to the nonlinear upconversion that is discussed in the main text.

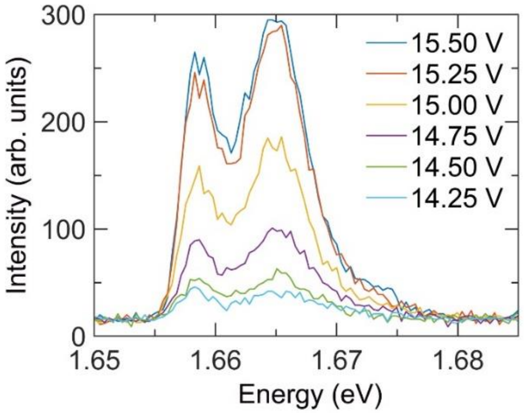

Fig. S2 Typical spectral profiles extracted from Fig. 3b. The intensity of the upconversion PL is sensitive to the resonance condition between trion state and cavity polaritons.

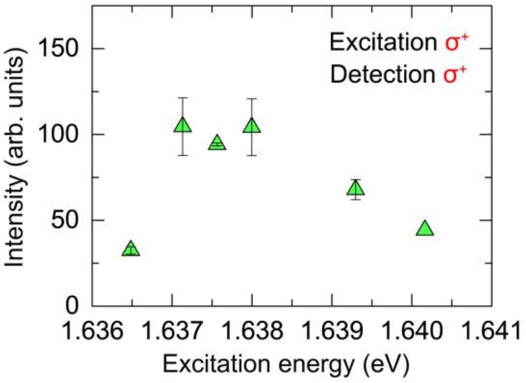

Fig. S3 Upconversion intensity at different excitation energies. Integrated intensity of upconversion PL under co-polarization conditions. We find a resonant feature where the laser energy matches the trion transition in spectra. Those measurements indicate that the upconversion is a resonant process, remarkably dependent on resonance transitions of TMDC monolayers. A similar phenomenon has been reported previously for the case of TMDC monolayers which are not coupled to optical cavities[1-3].

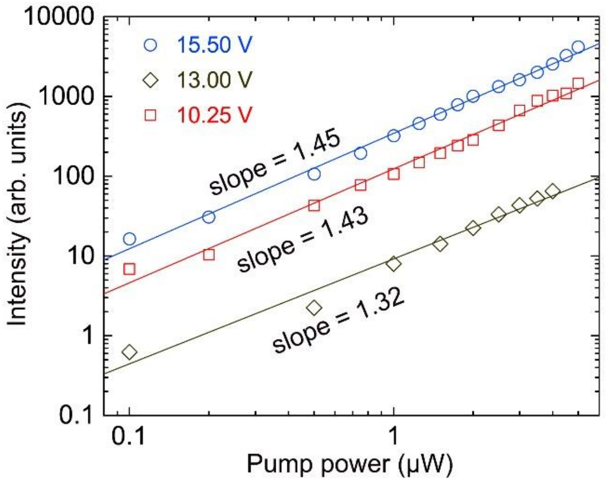

Fig. S4 Plotting of Fig. 4b with double logarithmic presentation. At 15.50 V and 10.25 V, where efficient upconversion luminescence occurs, the power-law coefficient is ~1.4. At 13.00 V, the pump laser is detuned from the polariton mode, and the intensity of upconversion luminescence is substantially reduced. A nonlinear scaling is also observed with a coefficient ~1.3.

Section 1. Hopfield model for exciton-trion-photon coupling

To better understand the character of the lower polariton at the trion energy, we use a Hopfield (three-oscillator) model to extract the Hopfield coefficients and distinguish the exciton and trion contributions, see Fig. S5. To obtain accurate coupling strengths, we take into account the effective DBR lengths[4] by calculating mirror reflectivities using the transfer-matrix method, and use experimentally extracted values for the exciton and trion oscillator strength. The Hopfield coefficients reveal that the exciton-trion mixing is weak, with a maximum exciton character of about 7%. In other words, the trion weakly couples to a very photon-like lower exciton-polariton branch. This is due to the large energy separation between the exciton and trion (32 meV) compared to the Rabi splitting (9 meV). Note that the trion-light coupling is too weak to show a Rabi splitting in both the experimental and calculated PL (see Fig. 2 in the main text).

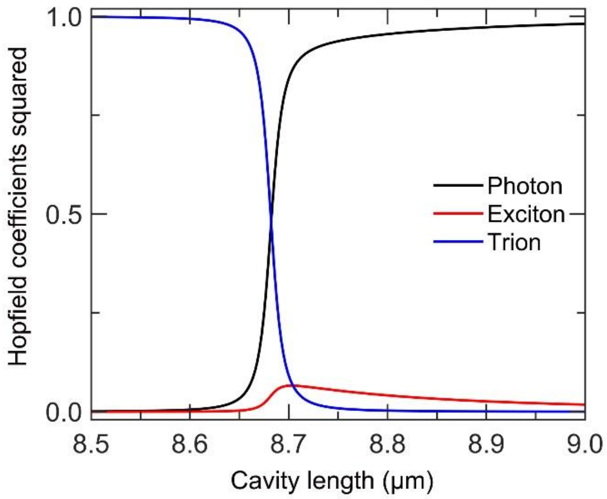

Fig. S5 Contributions to the lower polariton branch. Photonic (black), excitonic (red), and trionic (blue) contribution to the lower polariton, given by the square of the relevant Hopfield coefficient. The small exciton contribution reveals that the trion predominantly interacts with a bare cavity mode.

Section 2. Power dependent density ratio based on Saha equation

To estimate the thermalized population of exciton-polaritons, it is crucial to take into account the formation mechanism of the trion, and the role of the electron doping and pump power in driving the equilibrium ratio of trions and exciton-polaritons. For this, the mass action law can be used[5]. At high pump powers, trion generation will saturate and the generation of a substantial exciton polariton population can become favourable.

When calculating the ratio of the exciton and trion density, it is important to take into account the continuum of momentum states stemming from free charges in the $MoSe_2$ monolayer (donated from the GaInP layer). In the following, we model it with electron doping, while the model also applies for hole doping. In analogy to the ionization reaction of hydrogen, we can consider the formation of trions and excitons as a rate reaction: X^0 (excitons) + $e^- \leftrightharpoons X^-$(trions). Thus, the total densities of the free electrons (n_e), polaritons (n_P), and trions (n_T) are dictated by the Saha equation (mass action law) at steady state[5],

$$\frac{n_P n_e}{n_T} = 2\frac{Z_P}{Z_T} D_e k_B T \exp\left[-\frac{\left|\varepsilon^T\right| - g}{k_B T}\right] \tag{1}$$

where $Z_i = \sum_{\vec{k}} \exp[-\mathrm{E}_i(\vec{\mathrm{k}})/(\mathrm{k}_B \mathrm{T})]$ represents the partition function for the i-th constituent particle type, $D_\mathrm{e} = \frac{m_e}{\pi\hbar^2}$ the 2D density of states of a classical electron gas, and ε^T the trion binding energy[6], g the light-exciton coupling strength. The Saha equation must be complemented with constraints from particle and charge conservation

$$\begin{aligned} n_p &= n_P + n_T \\ n_d &= n_e + n_T \end{aligned} \tag{2}$$

Here, n_p is the number of photons absorbed by the pump laser, and n_d is the background doping concentration. Note that this model applies at thermal equilibrium, and the exact mechanisms for the upconversion do not need to be specified. For a large pump power, the reaction equilibrium can be shifted heavily in the favour of exciton-polariton formation, despite the 30 meV difference in energy, and low temperatures. This is shown in Fig. S6a. It can be understood as a consequence of trion saturation: each pump photon must generate an exciton or trion due to particle conservation, but when the free electron population is depleted due to a large existing trion population (see Fig. S6b), there is a preferential formation of exciton-polaritons. Note that quantum corrections (i.e., Pauli blocking) are expected at high electron densities and low temperatures. At the high-pump region of interest where the electron concentration drops to zero, we expect that the classical Saha equation should give qualitatively correct results.

The evaluation of the polariton partition function is performed numerically, but, for a low enough temperature ($k_B T \ll g$), the polariton population occupies states close to the polariton ground state and hence a parabolic approximation can be used. In this case, $\frac{Z_P}{Z_T} \approx \frac{M_P}{M_T}$, where M_P and M_T are the effective polariton and trion masses, respectively. This illustrates that the reduced polariton density of states will tend to reduce the fraction of exciton-polaritons ($M_P \sim 10^{-5} M_X$), but this can be compensated by the additional factor of $\exp(g/k_B T)$ accounting for the cavity-induced reduction in the ionization energy[6]. For the background electron concentration, we estimate 2×10^{12} cm^{-2} based on the trion binding energy[7]. To link the number of photons to the pump laser power, the average power of a pulsed laser beam is expressed

in terms of the fluence, $F = \frac{P_{avg}}{R\pi(d/2)^2}$ where R = 76.2 MHz is the repetition rate of the laser, and d ~ 3 μm is the diameter of beam waist. The photon concentration is connected to the fluence via $n_p = F/\hbar\omega$ where $\hbar\omega$ is the energy of the pump laser. This means that the power is related to the photon concentration via $P_{avg} = \hbar\omega n_p R\pi(d/2)^2$. Note that we have neglected the reflected portion of the incoming laser, meaning we overestimate n_p for a given average power.

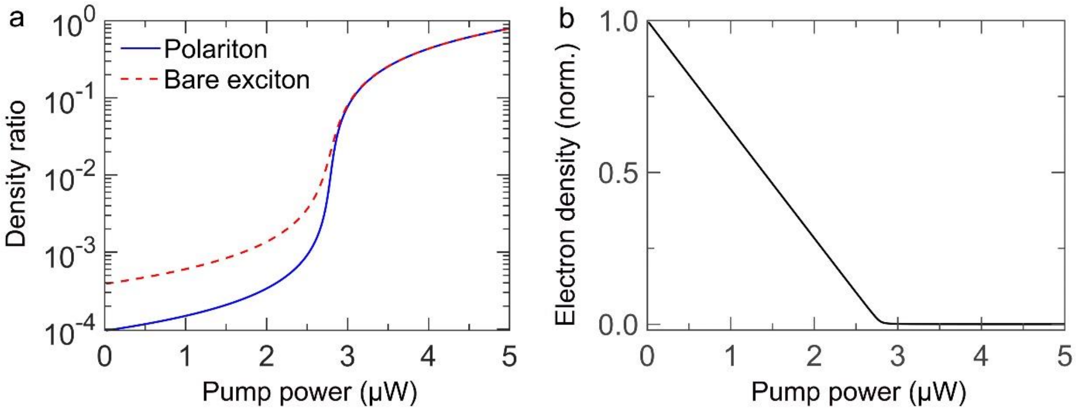

Fig. S6 Power dependent density ratio of Saha equation. a Ratio of the lower-energy exciton-polariton and trion concentration (blue curve) as a function of the laser power at 50 K. For comparison, we also show the ratio for the bare excitonic system without cavity (dashed red curve). **b** The corresponding free electron population normalized by the total doping concentration.

References

1. Jadczak, J., *et al.* Biexciton and Singlet Trion Upconvert Exciton Photoluminescence in a $MoSe_2$ Monolayer Supported by Acoustic and Optical K-Valley Phonons. *J. Phys. Chem. Lett.* **14**, 8702-8708 (2023).
2. Han, B., *et al.* Exciton States in Monolayer $MoSe_2$ and $MoTe_2$ Probed by Upconversion Spectroscopy. *Phys. Rev. X* **8**, 031073 (2018).
3. Manca, M., *et al.* Enabling valley selective exciton scattering in monolayer WSe_2 through upconversion. *Nat. Commun.* **8**, 14927 (2017).
4. Savona, V., Piermarocchi, C., Quattropani, A., Schwendimann, P., Tassone, F. Optical properties of microcavity polaritons. *Phase Transitions* **68**, 169-279 (1999).
5. Ross, J. S., *et al.* Electrical control of neutral and charged excitons in a monolayer semiconductor. *Nat. Commun.* **4**, 1474 (2013).
6. Fitzgerald, J. M., Thompson, J. J. P., Malic, E. Twist Angle Tuning of Moiré Exciton Polaritons in van der Waals Heterostructures. *Nano Lett.* **22**, 4468-4474 (2022).
7. Liu, E., *et al.* Exciton-polaron Rydberg states in monolayer $MoSe_2$ and WSe_2. *Nat. Commun.* **12**, 6131 (2021).
8. Lan, W., Wang, J., Xin, M., Huang, Y., Gu, C., Liu, B. Trion-to-exciton upconversion dynamics in monolayer WSe_2. *Appl. Phys. Lett.* **117**, 083107 (2020).
9. Hao, K., *et al.* Coherent and Incoherent Coupling Dynamics between Neutral and Charged Excitons in Monolayer $MoSe_2$. *Nano Lett.* **16**, 5109-5113 (2016).
10. Perea-Causín, R., *et al.* Exciton Propagation and Halo Formation in Two-Dimensional Materials. *Nano Lett.* **19**, 7317-7323 (2019).
